# Global, regional, and national burden of pulmonary arterial hypertension from 1990 to 2021

**DOI:** 10.1097/MD.0000000000044933

**Published:** 2025-10-10

**Authors:** Yunyan Lu, Jing Zhang, Tian Lan, Jiawei He

**Affiliations:** a Department of Cardiology, The First People’s Hospital of Xiaoshan District, Xiaoshan Affiliated Hospital of Wenzhou Medical University, Hangzhou, Zhejiang, People’s Republic of China; b Department of Breast Surgery, Hangzhou TCM Hospital Affiliated to Zhejiang Chinese Medical University, Hangzhou Hospital of Traditional Chinese Medicine, Hangzhou, Zhejiang, People’s Republic of China.

**Keywords:** epidemiology, global burden of disease, pulmonary arterial hypertension, time trends

## Abstract

Pulmonary arterial hypertension (PAH) is a life-threatening disorder characterized by pulmonary arterial remodeling. Limited quantitative research is available on the spatiotemporal burden of PAH. The prevalence, incidence, mortality, disability-adjusted life-years and corresponding age-standardized rates were derived from the global burden of disease 2021 study. Estimated annual percentage changes and average annual percentage changes were calculated to explore the disease trend. In 2021, there were approximately 1918,100 (95%UI: 1,553,600-2,357,900) prevalent cases of PAH worldwide, with an age-standardized prevalence rate (ASPR) of 2.28 (95%UI: 1.85–2.80) per 100,000 population. The number of incident cases amounted to about 432,500 (95%UI: 347,000–524,400), with an age-standardized incidence rate (ASIR) of 0.52 (95%UI: 0.42–0.62). It presented a stable status for ASPR, a slight increased trend for ASIR, a decline trend for age-standardized mortality rate and age-standardized disability-adjusted life-years rate during past 32 years. The countries with the highest ASPR were in Southern and Northern Europe. Meanwhile, the countries in Sub-Saharan Africa suffered highest ASIR, including Zambia, Ethiopia, Rwanda, and Malawi. The highest age-specific prevalence and incidence rates were observed in the 75 to 79 age group in 2021. The ASPR of PAH increased with the social-development index (SDI) across different countries, whereas the ASIR exhibited a negative correlation with SDI. The spatiotemporal burden of PAH revealed considerable geographic and demographic disparities from 1990 to 2021. Our findings could offer effective insights for shaping public health strategies and allocating health-care resources among regions with different SDI.

## 1. Introduction

Pulmonary arterial hypertension (PAH), 1 distinct subtype of pulmonary hypertension, is characterized by pulmonary artery pressure exceeding 20 mm Hg, pulmonary artery wedge pressure of 15 mm Hg or less, and pulmonary vascular resistance of 3 or more Wood units.^[1]^ Based on etiology, pathophysiology, and therapeutic response, PAH is classified into 7 distinct subgroups, such as idiopathic PAH, heritable PAH, drug-induced PAH, and others.^[2]^ Despite advancements in medication that have contributed to increased median survival, PAH remains an incurable and progressive cardiovascular disease which results in the structural alterations of the distal pulmonary vasculature and the infiltration of inflammatory cells.^[3]^ PAH detection has markedly risen, attributed to heightened awareness among health-care practitioners and advancements in diagnostic methodologies. PAH deserves recognition as a consequential global disease with a considerable prevalence that is largely overlooked.

Several epidemiological studies conducted in the United States, France, and Spain provide valuable insights into the national burden of PAH, unveiling 10.6 to 16 prevalent cases and 2 to 3.2 incident cases per 1000,000 population.^[4–6]^ The preceding understanding of PAH, derived from these cohort data encompassing a combination of incident and prevalent patients, may be subject to potential bias.^[7]^ These national studies in the developed countries do not fully elucidate the global distribution of PAH.^[8]^ Therefore, it is worthwhile to delve into the spatiotemporal trends of PAH on a global scale. Based on the Global Burden of Diseases (GBD), Injuries, and Risk Factors Study 2021, we explored the impact of geographical location, age, gender, and social-development index (SDI) on the spatiotemporal trends of PAH in terms of prevalence, incidence, deaths, and disability-adjusted life-years (DALYs).

## 2. Methods

### 2.1. Data acquisition and study population

The data pertaining to PAH was derived from the GBD 2021 study which provided a comprehensive estimation for 288 death causes and 88 risk factors across 204 countries and territories utilizing the advanced standardized methodologies and the latest epidemiological data.^[9]^ The numbers and rates of prevalence, incidence, death, and DALYs, along with the corresponding age-standardized rates (ASR) related to PAH, were obtain using the Global Health Data Exchange (GHDx) query tool (http://ghdx.healthdata.org/gbd-results-tool).

PAH in the GBD 2021 study was identified based on the disease codes I27.0 in the 10th revision of the International Classification of Diseases (ICD-10) or 416.0 in the previous version, ICD-9. The cause of death ensemble modelling, an effective statistical modeling approach, was used to estimate the deaths of PAH with several covariates. Disease Modeling Meta-Regression (DisMod-MR) version 2.1 was used in the GBD study to estimate PAH burden for all locations, including low-SDI countries. All measures (number, rate, and ASR) were estimated among different geographical regions and countries, encompassing twenty age groups for both women and men.

The SDI, which serves as a comprehensive index of the economic, social and health conditions in each country, ranges from 0 (worse) to 1 (best). Countries and territories were classified into 5 tiers, namely low, low-middle, middle, high-middle, and high SDI, based on SDI scores. Besides, they were divided into minimal, limited, basic, and advanced health systems. No ethics approval or consent to participate was necessary due to the absence of any individually identifiable information.

### 2.2. Statistical analysis

The estimations and their 95% uncertainty interval for all measures (number, rate, and ASR) were calculated from the GBD 2021 study. Uncertainty was quantified by sampling from the posterior distributions in the modeling process. All rates were reported per 100,000 population. For further evaluating the change in disease burden from 1990 to 2021, we calculated the estimated annual percentage change (EAPC) and average APC (AAPC) of ASRs. The equation Y = a + βX + ε represents a linear relationship between the natural logarithm of ASRs (Y) and the year (X). The EAPC was computed as 100 × (exp(β)-1). We calculated the APC and AAPC using the jpCommand software (Version 5.02) and the R package “nih.joinpoint.” The equation used to calculate AAPC was set as followed.


$$\begin{array}{l} AAPC=\left\{exp\left(\frac{\sum w_{i}b_{i}}{\sum w_{i}}\right)\right\}\times 100 \end{array}$$

Pearson correlation analysis was performed to assess the relationship between ASRs and SDI in 2021. All visualization and statistical analysis were performed by R (version 4.2.3). A *P*-value of <0.05 was deemed statistically significant.

## 3. Results

### 3.1. The global burden of PAH

**Figure 1.**
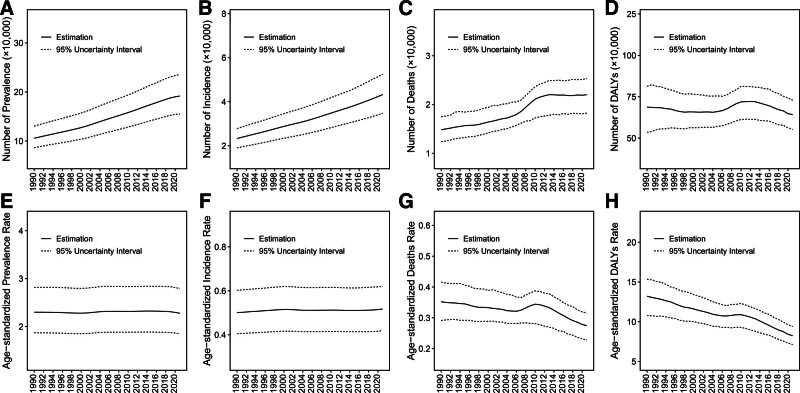
The change in the cases and corresponding age-standardized rates of PAH, 1990 to 2021. The number of prevalence (A), incidence (B), death (C), and DALYs (D). The age-standardized rate of prevalence (E), incidence (F), death (G), and DALYs (H). DALYs = disability-adjusted life-years, PAH = pulmonary arterial hypertension.

**Figure 2.**
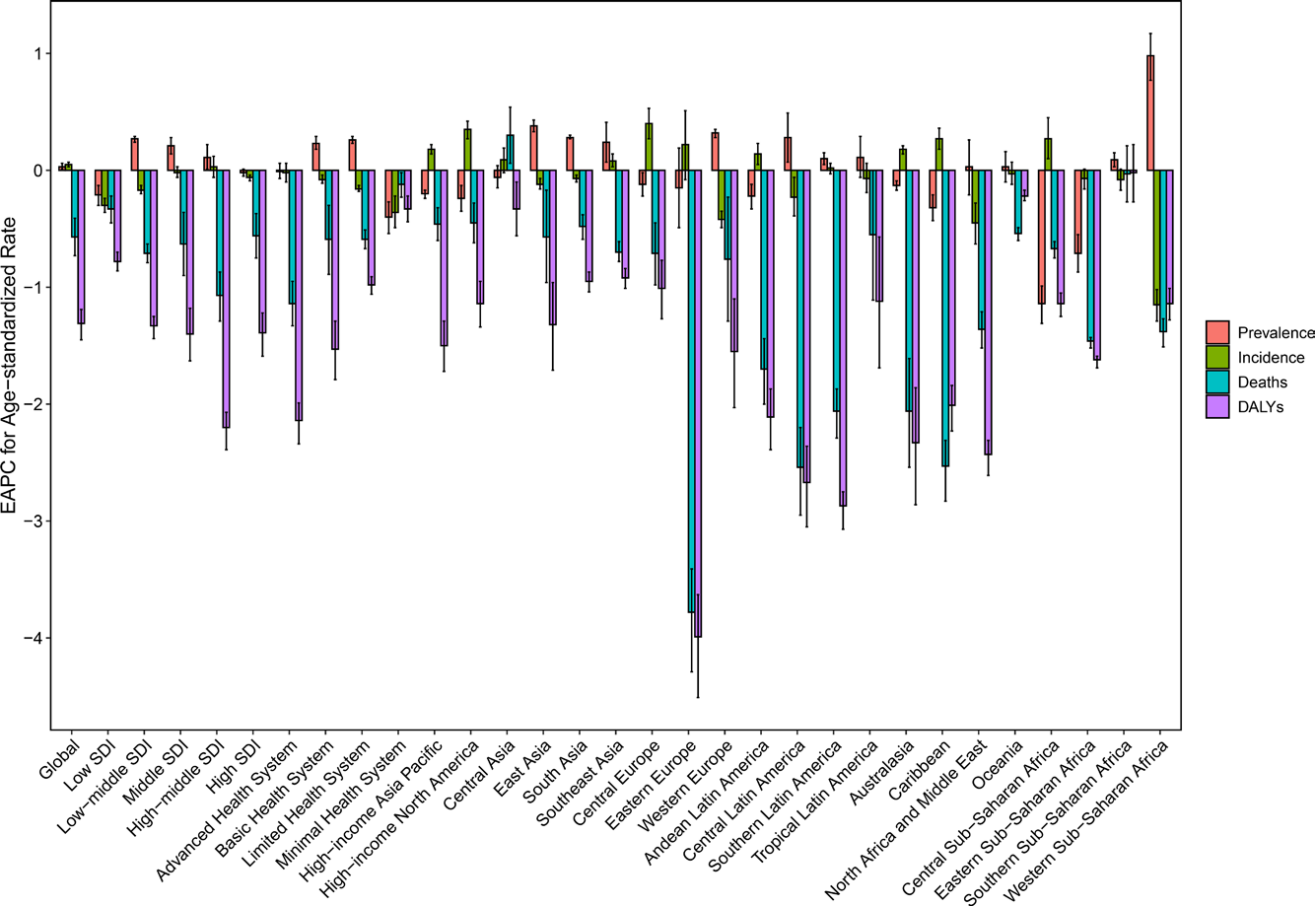
Estimated annual percentage change of the age-standardized prevalence, incidence, death, and DALYs rates across different SDI regions, health systems, and GBD regions. DALYs = disability-adjusted life-years, GBD = Global Burden of Disease, SDI = socio-demographic index.

In 2021, there were approximately 1918,100 (95%UI: 1,553,600–2,357,900) prevalent cases of PAH worldwide, with an age-standardized prevalence rate (ASPR) of 2.28 (95%UI: 1.85–2.80) per 100,000 population (Table 1 and Table S1, Supplemental Digital Content, https://links.lww.com/MD/Q259). The number of incident cases amounted to about 432,500 (95%UI: 347,000–524,400), with an age-standardized incidence rate (ASIR) of 0.52 (95%UI: 0.42–0.62). The number of prevalence and incidence showed a significant increase (Fig. 1A and B). However, it presented a relative stable trend for ASPR based on the EAPC (0.03, 95% CI: 0.01 to 0.06) and AAPC (−0.03, 95% CI: −0.04 to −0.02) (Figs. 1E and 2, and Tables S2 and S3, Supplemental Digital Content, https://links.lww.com/MD/Q259). A marginal rise was observed in ASIR, as evidenced by the positive values of EAPC (0.05, 95% CI: 0.03 to 0.07) and AAPC (0.10, 95% CI: 0.10 to 0.11) (Fig. 1F and 2, and Tables S2 and S3, Supplemental Digital Content, https://links.lww.com/MD/Q259).

**Table 1:** The age-standardized rates of prevalence, incidence, death, and DALYs for PAH in 1990 and 2021.

	Age-standardized prevalence rate		Age-standardized incidence rate		Age-standardized death rate		Age-standardized DALYs rate	
Location	1990	2021	1990	2021	1990	2021	1990	2021
Global	2.30 (1.87–2.82)	2.28 (1.85–2.80)	0.50 (0.40–0.60)	0.52 (0.42–0.62)	0.35 (0.29–0.42)	0.27 (0.23–0.32)	13.21 (10.78–15.36)	8.24 (7.14–9.39)
Male	1.75 (1.42–2.13)	1.78 (1.44–2.17)	0.47 (0.38–0.57)	0.48 (0.39–0.58)	0.37 (0.29–0.46)	0.27 (0.21–0.33)	14.09 (11.89–16.35)	8.06 (6.72–9.36)
Female	2.81 (2.29–3.46)	2.75 (2.24–3.39)	0.53 (0.43–0.63)	0.55 (0.45–0.66)	0.34 (0.23–0.45)	0.28 (0.22–0.34)	12.32 (7.78–16.80)	8.39 (6.92–10.53)
Low SDI	2.08 (1.68–2.53)	1.94 (1.58–2.36)	0.78 (0.64–0.93)	0.71 (0.58–0.85)	0.32 (0.15–0.51)	0.27 (0.15–0.40)	12.42 (7.78–19.19)	9.30 (6.08–13.20)
Low-middle SDI	1.77 (1.43–2.15)	1.90 (1.53–2.33)	0.60 (0.48–0.71)	0.59 (0.47–0.70)	0.35 (0.22–0.47)	0.26 (0.18–0.38)	14.92 (10.74–18.41)	9.07 (7.05–11.60)
Middle SDI	2.07 (1.68–2.54)	2.21 (1.80–2.71)	0.54 (0.43–0.64)	0.53 (0.43–0.64)	0.45 (0.35–0.58)	0.33 (0.22–0.39)	14.29 (11.66–17.69)	8.23 (6.26–9.70)
High-middle SDI	2.61 (2.12–3.21)	2.54 (2.07–3.12)	0.43 (0.35–0.52)	0.46 (0.37–0.55)	0.35 (0.31–0.43)	0.24 (0.20–0.29)	13.14 (10.91–16.04)	6.48 (5.61–7.87)
High SDI	2.67 (2.17–3.27)	2.64 (2.15–3.23)	0.37 (0.30–0.45)	0.37 (0.29–0.44)	0.26 (0.24–0.28)	0.22 (0.19–0.23)	9.16 (8.71–9.90)	6.16 (5.76–6.49)
Advanced Health System	2.80 (2.27–3.43)	2.73 (2.22–3.35)	0.37 (0.30–0.45)	0.38 (0.31–0.47)	0.30 (0.26–0.33)	0.22 (0.19–0.23)	12.02 (9.89–13.92)	6.47 (6.09–6.86)
Basic Health System	2.13 (1.73–2.61)	2.26 (1.83–2.77)	0.52 (0.42–0.63)	0.51 (0.41–0.62)	0.46 (0.36–0.60)	0.33 (0.25–0.40)	15.69 (12.75–19.50)	8.61 (6.83–10.56)
Limited Health System	1.77 (1.44–2.16)	1.91 (1.54–2.33)	0.63 (0.51–0.75)	0.61 (0.49–0.73)	0.31 (0.17–0.45)	0.24 (0.16–0.37)	11.83 (8.04–15.39)	8.30 (6.15–10.94)
Minimal Health System	2.30 (1.86–2.80)	1.94 (1.58–2.34)	0.83 (0.68–0.99)	0.74 (0.60–0.89)	0.28 (0.12–0.56)	0.25 (0.12–0.43)	11.12 (6.51–19.43)	9.29 (5.19–14.76)
High-income Asia Pacific	3.25 (2.67–3.97)	3.01 (2.47–3.68)	0.31 (0.25–0.37)	0.33 (0.26–0.40)	0.26 (0.24–0.27)	0.23 (0.20–0.26)	12.03 (11.43–12.79)	8.31 (7.74–8.87)
High-income North America	1.87 (1.53–2.29)	1.73 (1.40–2.12)	0.27 (0.22–0.33)	0.30 (0.24–0.37)	0.32 (0.28–0.35)	0.29 (0.26–0.31)	10.13 (9.39–10.99)	7.71 (7.17–8.18)
Central Asia	2.46 (2.01–3.00)	2.33 (1.90–2.85)	0.41 (0.33–0.50)	0.44 (0.36–0.54)	0.40 (0.31–0.47)	0.41 (0.34–0.48)	14.14 (11.27–16.45)	12.91 (10.61–15.60)
East Asia	2.07 (1.68–2.55)	2.23 (1.81–2.75)	0.51 (0.41–0.61)	0.50 (0.40–0.60)	0.59 (0.45–0.81)	0.41 (0.28–0.50)	15.78 (12.34–21.21)	8.84 (5.99–11.01)
South Asia	1.58 (1.28–1.93)	1.71 (1.38–2.09)	0.56 (0.46–0.67)	0.56 (0.45–0.67)	0.31 (0.17–0.50)	0.25 (0.16–0.42)	12.24 (7.79–17.12)	8.54 (6.02–12.46)
Southeast Asia	1.77 (1.43–2.17)	1.90 (1.54–2.32)	0.55 (0.44–0.66)	0.58 (0.47–0.69)	0.15 (0.09–0.43)	0.12 (0.08–0.32)	6.31 (4.28–12.72)	4.65 (3.39–9.25)
Central Europe	2.52 (2.08–3.05)	2.30 (1.89–2.79)	0.39 (0.31–0.47)	0.47 (0.38–0.57)	0.26 (0.22–0.28)	0.21 (0.19–0.23)	8.15 (7.25–8.95)	6.05 (5.50–6.67)
Eastern Europe	3.21 (2.65–3.91)	2.83 (2.32–3.43)	0.37 (0.30–0.45)	0.43 (0.35–0.52)	0.24 (0.22–0.27)	0.09 (0.08–0.10)	9.23 (8.47–10.49)	3.32 (3.10–3.56)
Western Europe	3.22 (2.62–3.95)	3.56 (2.92–4.35)	0.46 (0.37–0.55)	0.41 (0.33–0.49)	0.24 (0.21–0.27)	0.18 (0.16–0.19)	8.27 (7.69–9.18)	5.05 (4.75–5.32)
Andean Latin America	2.91 (2.35–3.58)	2.77 (2.24–3.40)	0.53 (0.43–0.63)	0.55 (0.44–0.66)	0.28 (0.21–0.35)	0.16 (0.12–0.20)	11.92 (7.83–16.43)	5.73 (4.52–7.28)
Central Latin America	2.71 (2.21–3.30)	3.25 (2.64–3.99)	0.56 (0.46–0.68)	0.49 (0.40–0.59)	0.16 (0.14–0.19)	0.08 (0.07–0.10)	6.12 (5.40–7.16)	3.00 (2.63–3.51)
Southern Latin America	2.69 (2.18–3.30)	2.82 (2.29–3.48)	0.34 (0.28–0.41)	0.33 (0.27–0.41)	0.36 (0.32–0.40)	0.18 (0.17–0.20)	16.28 (14.65–17.95)	6.55 (6.13–7.07)
Tropical Latin America	2.35 (1.90–2.88)	2.48 (2.00–3.04)	0.50 (0.40–0.60)	0.49 (0.39–0.59)	0.37 (0.35–0.39)	0.32 (0.29–0.34)	14.18 (13.35–14.98)	10.22 (9.65–10.78)
Australasia	2.99 (2.44–3.70)	2.83 (2.29–3.44)	0.35 (0.28–0.43)	0.37 (0.30–0.45)	0.21 (0.18–0.26)	0.11 (0.10–0.13)	7.37 (6.49–9.06)	3.67 (3.38–3.99)
Caribbean	2.56 (2.08–3.13)	2.39 (1.93–2.93)	0.45 (0.37–0.55)	0.48 (0.39–0.58)	0.38 (0.28–0.49)	0.20 (0.13–0.29)	19.72 (11.78–28.82)	11.73 (6.48–18.74)
North Africa and Middle East	2.02 (1.64–2.49)	2.03 (1.64–2.49)	0.56 (0.45–0.67)	0.52 (0.42–0.63)	0.77 (0.56–1.00)	0.44 (0.31–0.53)	35.84 (21.25–46.14)	14.81 (10.76–17.96)
Oceania	1.98 (1.60–2.40)	1.80 (1.45–2.17)	0.57 (0.46–0.68)	0.61 (0.49–0.73)	0.28 (0.18–0.53)	0.24 (0.16–0.48)	10.85 (7.02–17.55)	10.14 (6.90–17.10)
Central Sub-Saharan Africa	2.79 (2.26–3.44)	1.86 (1.49–2.24)	0.81 (0.65–0.97)	0.83 (0.67–0.98)	0.24 (0.11–0.47)	0.19 (0.08–0.37)	9.09 (5.65–17.13)	6.16 (2.94–11.09)
Eastern Sub-Saharan Africa	2.40 (1.94–2.92)	2.09 (1.70–2.53)	0.99 (0.81–1.17)	0.92 (0.75–1.09)	0.27 (0.12–0.52)	0.18 (0.07–0.34)	11.11 (6.18–21.17)	6.85 (3.30–12.62)
Southern Sub-Saharan Africa	2.12 (1.73–2.56)	2.28 (1.85–2.76)	0.81 (0.67–0.97)	0.75 (0.61–0.90)	0.12 (0.08–0.17)	0.11 (0.08–0.13)	4.70 (3.65–6.37)	4.33 (3.21–5.20)
Western Sub-Saharan Africa	2.42 (1.96–2.97)	2.97 (2.40–3.63)	0.83 (0.68–0.99)	0.64 (0.52–0.78)	0.25 (0.09–0.51)	0.17 (0.07–0.28)	9.34 (5.22–16.94)	6.50 (3.77–9.47)

DALYs = disability-adjusted life-years, PAH = pulmonary arterial hypertension, SDI = socio-demographic index.

The number of deaths attributed to PAH rose from 148,400 (95%UI: 123,700–174,800) in 1990 to 220,200 (95%UI: 182,400–253,500) in 2021. However, the age-standardized death rate (ASMR) demonstrated a decline, with EAPC at −0.57 (95% CI: −0.73 to −0.41) and AAPC at −0.84 (95% CI: −0.87 to −0.82) (Figs. 1C, 1G, 2, and Table 1, Tables S1–S3, Supplemental Digital Content, https://links.lww.com/MD/Q259). Meanwhile, the DALYs cases decreased from 6874,200 (95%UI: 5352,400-8130,900) in 1990 to 6421,000 (95%UI: 5522,700-7289,900) in 2021, accompanied by a decline in age-standardized DALYs rate (ASDR) due to EAPC (−1.31, 95% CI: −1.45 to −1.19) and AAPC (-1.54, 95% CI: −1.56 to −1.52) (Figs. 1D, 1H, 2, and Table 1, Tables S1–S3, Supplemental Digital Content, https://links.lww.com/MD/Q259).

### 3.2. The reginal burden of PAH

In all 5 SDI regions, the prevalent and incident cases of PAH exhibited a prominent escalation during 1990–2021 (Table S1, Supplemental Digital Content, https://links.lww.com/MD/Q259). The highest number of deaths and DALYs were observed in the middle SDI region. The ASPR increased along with SDI growth in 2021, yet the ASIR and ASDR decreased (Table 1). Based on consistent findings from EAPC and AAPC, there was a modest upsurge of ASPR in the regions with low-middle and middle SDI (Fig. 2, Fig. S1A, Supplemental Digital Content, https://links.lww.com/MD/Q258, Tables S2 and S3, Supplemental Digital Content, https://links.lww.com/MD/Q259). It presented a stable status of ASIR and a downward trend of ASMR and ASDR across all SDI regions (Fig. S1B–D, Supplemental Digital Content, https://links.lww.com/MD/Q258).

In 2021, the highest cases of prevalence, incidence, death, and DALYs were identified in the basic health system (Table S1, Supplemental Digital Content, https://links.lww.com/MD/Q259). As the health system underwent enhancements, the ASPR increased, while the ASIR gradually decreased. The basic health system had the highest ASMR, whereas the minimal health system showed the highest ASDR. There was a substantial rise of ASPR in the limited health-care systems (Fig. S2A, Supplemental Digital Content, https://links.lww.com/MD/Q258). Across all health systems, the ASIR remained stable from 1990 to 2021, while both ASMR and ASDR exhibited a downward trend (Fig. S2B–D, Supplemental Digital Content, https://links.lww.com/MD/Q258). The reduction in the APC of ASIR increased with the deterioration of healthy system, while that of ASDR demonstrated the opposite pattern (Fig. 2).

At the GBD regional level, Eastern Asia had the highest cases of prevalence (424,900, 95%UI: 339,300–530,400), incidence (95,700, 95%UI: 76,000–119,000), death (74,900, 95%UI: 49,900–92,700), and DALYs (1,547,400, 95%UI: 1,029,400-1904,000) in 2021 (Table S1, Supplemental Digital Content, https://links.lww.com/MD/Q259). In addition, Western Europe showed the higher ASPR (3.56, 95%UI: 2.92–4.35) than other GBD regions (Table 1). Our findings indicated sub-Saharan African remained the region with the highest ASIR. The highest ASMR and ASDR were observed in North Africa and Middle East. According to the EAPC and AAPC, there was an upward trend for ASPR in East Asia, South Asia, Southeast Asia, Western Europe, Central Latin America, Southern Latin America, Southern Sub-Saharan Africa, and Western Sub-Saharan Africa. The highest EAPC (0.40, 95% CI: 0.27– 0.53) and AAPC (0.58, 95% CI: 0.56–0.61) of ASIR were observed in Central Europe. In all GBD regions, except for Central Asia, the EAPCs of ASMR and ASDR were negative values, indicating a downward trend. Moreover, we presented the lowest EAPC and AAPC of ASMR and ASDR in Easten Europe.

### 3.3. The national burden of PAH

**Figure 3.**
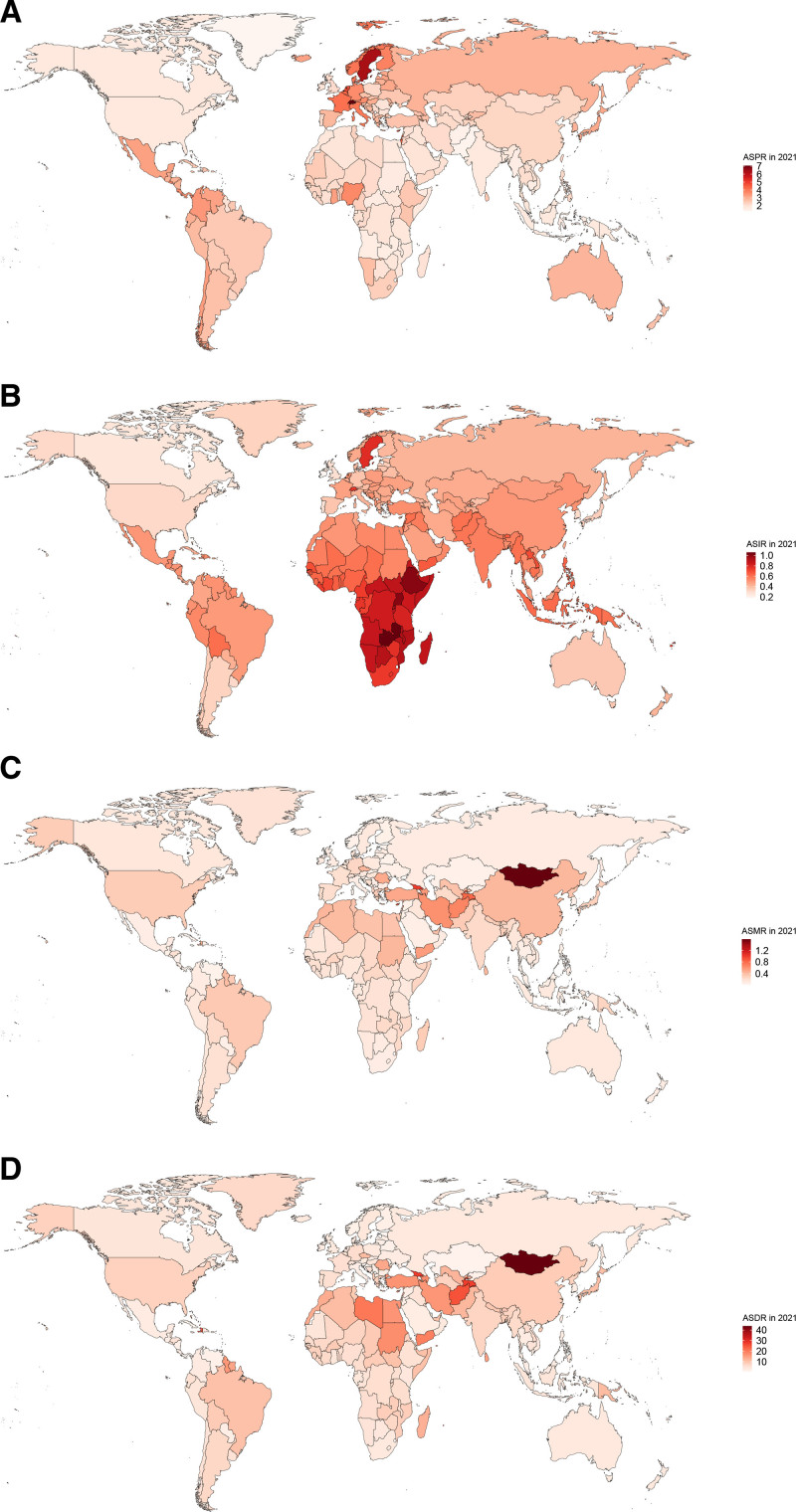
Global distribution of age-standardized rates for PAH in 2021. The age-standardized prevalence rate (A), The age-standardized incidence rate (B), The age-standardized death rate (C), The age-standardized DALYs rate (D). DALYs = disability-adjusted life-years, PAH = pulmonary arterial hypertension.

The global distribution of the prevalent, incident, death, DALYs cases were shown in Figure S3A–D, Supplemental Digital Content, https://links.lww.com/MD/Q258, and Table S4, S5, Supplemental Digital Content, https://links.lww.com/MD/Q259. China ranked first in terms of both prevalence and incidence cases, followed by India, the United States of America, and Brazil (Fig. S4A and B, Supplemental Digital Content, https://links.lww.com/MD/Q258). The top 3 countries with the highest death and DALYs cases were China, India, and the United States of America (Fig. S4C and D, Supplemental Digital Content, https://links.lww.com/MD/Q258). The countries with the highest ASPR were in Northern Europe (Switzerland, Sweden, Denmark, and Norway) and Southern Europe (France and Italy) (Fig. 3A, Fig. S5A, Supplemental Digital Content, https://links.lww.com/MD/Q258). Meanwhile, the countries in Sub-Saharan Africa suffered highest ASIR, including Zambia, Ethiopia, Rwanda, and Malawi (Fig. 3B, Fig. S5B, Supplemental Digital Content, https://links.lww.com/MD/Q258). The worldwide distribution of ASMR closely resembled that of ASDR (Figs. 3C, 3D). Specifically, Mongolia, Georgia, and Tajikistan had highest ASMR and ASDR (Fig. S5C and D, Supplemental Digital Content, https://links.lww.com/MD/Q258).

**Figure 4.**
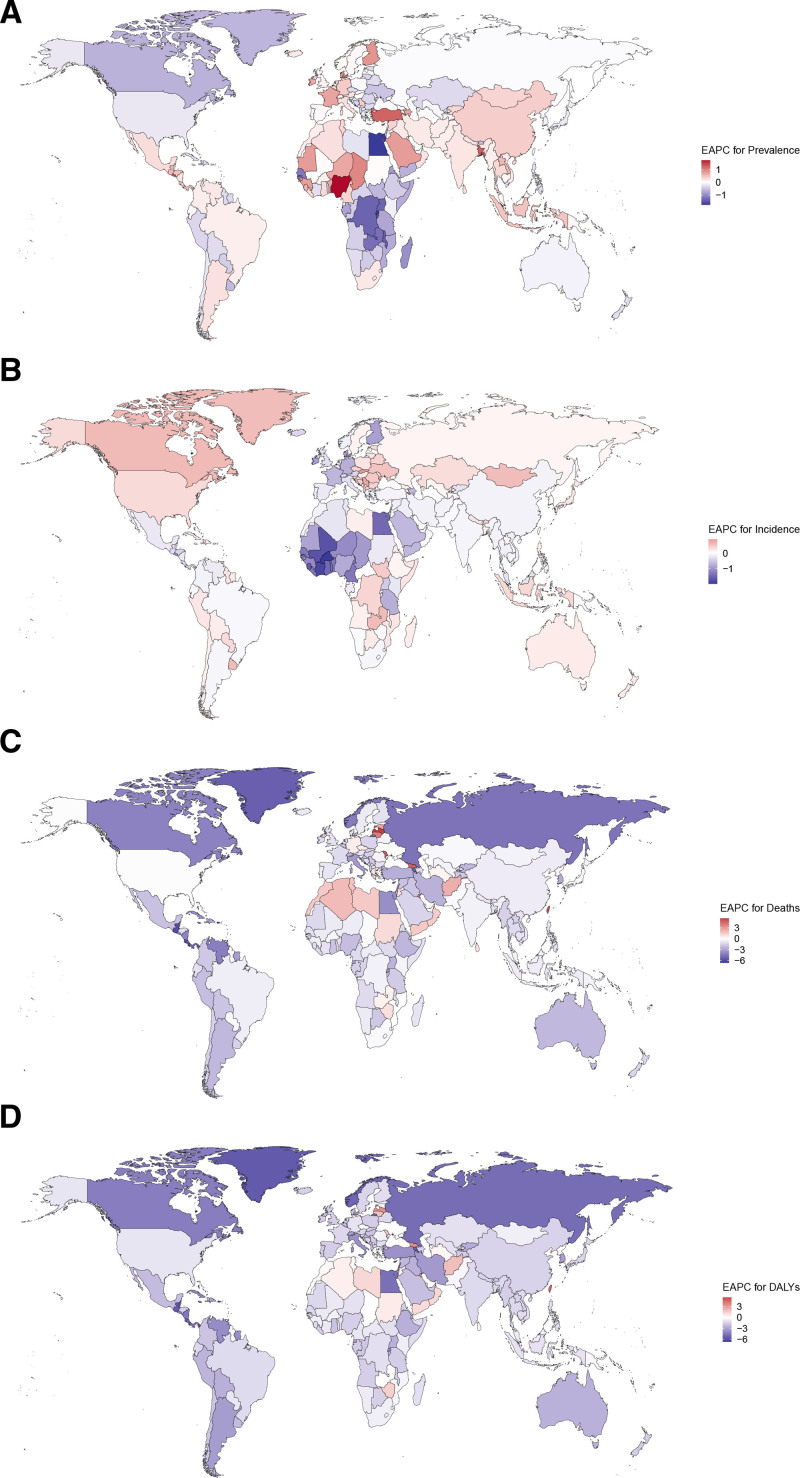
Global distribution of EAPC for PAH. The EAPC of prevalence (A), incidence (B), death (C), and DALYs (D). DALYs = disability-adjusted life-years, EAPC = estimated annual percentage change, PAH = pulmonary arterial hypertension.

From 1990 to 2021, the countries with the largest increases in ASPR were Nigeria, EI Salvador, Bangladesh, and Türkiye (Fig. 4A, Fig. S6A, Supplemental Digital Content, https://links.lww.com/MD/Q258). Eastern Europe (Slovakia, Serbia, Ukraine, and Albania) and Northern America (Canada and Greenland) exhibited the most significant growth in ASIR (Fig. 4B, Fig. S6B, Supplemental Digital Content, https://links.lww.com/MD/Q258). The EAPCs of ASMR and ASDR shared the similar global distribution (Figs. 4C, 4D). In the majority of countries worldwide, there was a downward trend observed for both ASMR and ASDR. However, notable upward trends were observed in Latvia, Taiwan (Province of China), Georgia, and Lithuania (Fig. S6C and D, Supplemental Digital Content, https://links.lww.com/MD/Q258)

### 3.4. Age, sex and SDI patterns

Globally, the highest prevalent and incident cases of PAH was observed in the age group of 55 to 70 in 2021 (Fig. S7, Supplemental Digital Content, https://links.lww.com/MD/Q258). The mortality count was notably higher in 2 specific age groups, namely 0 to 5 and 80 to 84. As age advanced, there was a discernible escalation in the age-specific rates of prevalence and incidence, culminating in the peak within the 75 to 79 age (Fig. S8, Supplemental Digital Content, https://links.lww.com/MD/Q258). In individuals aged more than 5, it revealed a noticeable rise in the age-specific death and DALYs rates, corresponding to the increase in age. The children aged less than 5 experienced a comparatively higher rate of DALYs. In addition, the age-specific EAPCs of ASPR remained relatively stable within the 0 to 85 age group and increased dramatically among individuals aged over 85 (Fig. S9A, Supplemental Digital Content, https://links.lww.com/MD/Q258). The EAPCs of ASIR among individual aged over 50 were lower than those among population aged under 50 (Fig. S9B, Supplemental Digital Content, https://links.lww.com/MD/Q258). A similar pattern in the EAPCs of ASMR and ASDR was evident in Figures S9C and D, Supplemental Digital Content, https://links.lww.com/MD/Q258.

In 2021, the age-specific cases of prevalence, incidence, and death in female were higher than that in male (Fig. S7, Supplemental Digital Content, https://links.lww.com/MD/Q258). The disparity of age-specific cases between female and male in 2021 was greater than that in 1990. Gender differences in age-specific rates were only evident in prevalence, not in other measures (Fig. S8, Supplemental Digital Content, https://links.lww.com/MD/Q258). Among the population aged from 25 to 90, the age-specific EAPCs of ASPR in female was apparently lower than that in male (Fig. S9A, Supplemental Digital Content, https://links.lww.com/MD/Q258). On the contrary, the age-specific EAPCs of ASIR in the female population aged over 70 were higher compared to those in the corresponding male population (Fig. S9B, Supplemental Digital Content, https://links.lww.com/MD/Q258). For ASMR and ASDR, the female EAPCs surpassed slightly those in male (Fig. S9C and D, Supplemental Digital Content, https://links.lww.com/MD/Q258).

**Figure 5.**
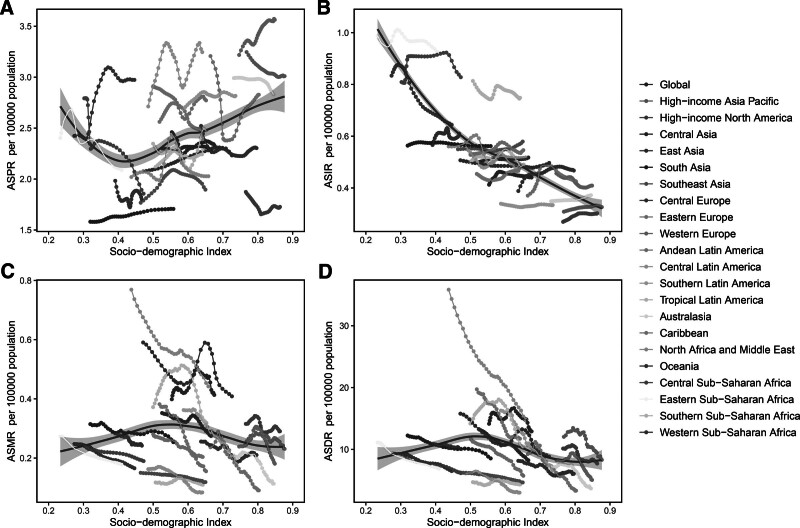
The trends in age-standardized rates of prevalence (A), incidence (B), death (C), and DALYs (D) for PAH across 21 GBD regions by SDI. DALYs = disability-adjusted life-years, GBD = Global Burden of Disease, PAH = pulmonary arterial hypertension, SDI = socio-demographic index.

For the total population, there was an upward trend of ASPR in the low-middle, middle, and high-middle SDI regions (Fig. S10A, Supplemental Digital Content, https://links.lww.com/MD/Q258). The gender difference in ASIR decreased with the SDI improvement (Fig. S10B, Supplemental Digital Content, https://links.lww.com/MD/Q258). The semblable change trends of ASMR and ASDR among different SDI and gender were shown in Figure S10C and D, Supplemental Digital Content, https://links.lww.com/MD/Q258. Additionally, a “V”-shaped curve was observed to depict the relationship between ASPR and SDI (Fig. 5A). Among the 21 GBD regions, ASIR decreased with SDI growth (Fig. 5B). ASMR and ASDR were higher than the expected level in several regions, including North Africa and Middle East, Central Asia, and Tropical Latin America (Fig. 5C and D).

**Figure 6.**
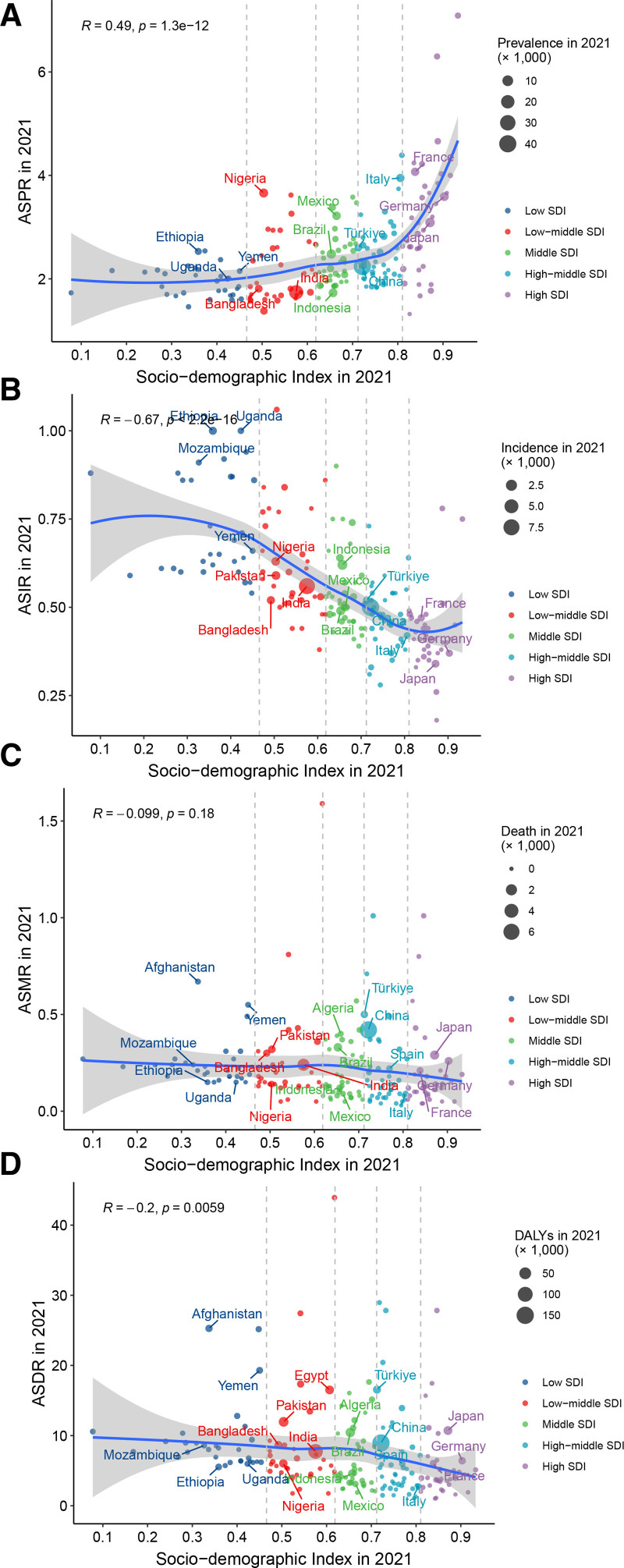
The age-standardized prevalence (A), incidence (B), death (C), and DALYs (D) rates of PAH across 204 countries and territories by SDI. DALYs = disability-adjusted life-years, PAH = pulmonary arterial hypertension, SDI = socio-demographic index.

A positive correlation was identified between ASPR and SDI in 2021 across 204 countries or territories (*R* = 0.49, *P* < .01), which was particularly pronounced in countries with SDI more than 0.8 (Fig. 6A). However, as depicted in Figure 6B, there was a negative relationship between ASIR and SDI in 2021 (R = -0.67, *P* < .01). It presented a flat curve for the association between ASMR, ASDR and SDI (Fig. 6C and D). We also explored a comparable relationship between EAPCs and ASD in 2021 (Fig. S11, Supplemental Digital Content, https://links.lww.com/MD/Q258). In the countries where SDI exceeds 0.8, it revealed a positive correlation between EAPC of ASPR and SDI.

## 4. Discussion

In this study, we conducted a comprehensive and systematic analysis of the epidemiological trends of PAH utilizing the latest GBD 2021 data. We revealed 1.92 million prevalent cases, 0.42 million incident cases, 0.22 million deaths, and 6.42 million DALYs of PAH globally in 2021. While it presented a stable status for ASPR, a decline trend for ASMR and ASDR, a slight increased trend for ASIR during past 32 years. These shifts can be partly attributed to the widespread use of echocardiography and the availability of innovative medications.^[10]^ We demonstrated that the highest age-specific rates of prevalence and incidence were observed in the age group of 75 to 79 in 2021. On the contrast, the median age of patients diagnosed with PAH was approximately 50 years in several registry cohorts.^[4–6]^ Furthermore, our study revealed the female predominance in the prevalence and incidence, aligning with prior researches.^[11]^ The age-specific EAPCs in male prevalence were higher than that in female.

There was a decline in the age-specific DALYs rate among individuals aged less than 5 from 1990 to 2021. However, the rate remained relatively high in 2021. The etiological distribution in pediatric PAH differed significantly from that observed in adults.^[12]^ It is imperative to require some pediatric-specific clinical trials in order to develop tailored treatment and decline the PAH burden in these children with PAH.

The ASPR of PAH increased by SDI across different countries. Conversely, the ASIR was found to be negatively correlated with SDI. Several plausible explanations can be attributed to these phenomena. First, PAH is an ailment that incurs substantial financial burden. The average health-care expenses associated with PAH ranged from $2023 to $9295 per patient per month.^[13,14]^ Second, in the countries with lower SDI, more patients with rapidly or severe PAH may die early because of exorbitant cost and insufficient medical support.^[15,16]^ Third, the underdiagnosis rate of PAH was found to be higher in nations with lower SDI compared to those with higher SDI, which may be caused by lack of standardized definition and diagnosis methods.^[16]^ Fourth, the individuals with a prolonged period of stable disease experienced improved survival rates and may receive more effective medical care in the countries with higher SDI.^[7]^

In 2021, a comparatively higher ASIR was noted in the countries of Sub-Saharan Africa, which may be attributed to the specific definition and classification of PAH established in 2019.^[17]^ Accessibility of echocardiography and pulmonary function testing in Sub-Saharan Africa was helpful to confirm the diagnosis and classification of PAH for clinicians. In addition, HIV and schistosomiasis were recognized as 2 etiological factors contributing to PAH.^[18]^ It was estimated that 25.5 million individuals were living with HIV in Sub-Saharan Africa.^[19]^ Schistosomiasis mansoni was endemic in Sub-Saharan Africa, exhibiting significant morbidity and prevalence.^[20]^ Therefore, the elevated incidence of HIV and schistosomiasis may contribute to a higher ASIR of PAH, including PAH associated with schistosomiasis or HIV.

Although ASMR and ASDR declined globally during 1990 to 2021, North Africa and Central Asia suffered relatively higher ASMR and ASDR. Mongolia, Georgia, and Tajikistan had highest ASMR and ASDR. In these regions and countries, the promotion and widespread adoption of PAH risk assessments based on the ESC-ERS (European Society of Cardiology–European Respiratory Society) risk score and the REVEAL (Registry to Evaluate Early and Long-Term PAH Disease Management) risk score should be encouraged.^[21,22]^ The 3 aberrant pathways of endothelin-1, prostacyclin, and nitric oxide were associated with endothelial dysfunction in PAH.^[23]^ The administration, cost and availability of the current developed targeted drugs correcting these signaling pathways were the major limiting factors in the PAH treatment.^[24]^ These medications remained prohibitively expensive for the average patient in regions with low SDI. A collaborative evaluation of cost, risk, and benefit by clinicians, patients, and their families may serve as a practical approach for patients in these countries. The government should implement measures to enhance the affordability and accessibility of these medications.

Several inevitable limitations should be considered when interpreting our findings. First, the PAH is not a single disease but rather a comprehensive group of diseases, including idiopathic PAH, heritable PAH, drug- and toxin induced PAH, and among others.^[25]^ Due to the limited availability of data, we excluded these characteristics from our analysis. Second, the data sources with more than 50% completeness were included in the GBD study, drawn from vital registration, verbal autopsy, registry, and surveillance data.^[26]^ The sparsity or unreliability of data from specific countries or territories that are not members of the World Health Organization may impact the accuracy of our findings. Third, the inconsistent PAH detection and diagnosis across different countries and time periods may compromise the comparability of the results. Despite these limitations, we expect that the epidemiological findings regarding PAH will provide more timely and actionable insights for shaping policy strategies and public health plans.

## 5. Conclusions

In summary, we outlined the spatiotemporal burden of PAH on a global, regional, and national scale by using the latest GBD 2021 study. We presented a declining trend in ASMR and ASDR, a slight increase in ASIR, and stable ASPR globally during past 32 years. The ASPR positively correlated with the SDI across countries, while the ASIR exhibited a negative correlation with SDI. These epidemiological findings highlight the need for enhancements in public health policymaking, diagnosis, treatment, and management of PAH, as well as the rational allocation of medical resources.

## Acknowledgments

We acknowledge The GBD, Injuries, and Risk Study 2021, which presented detailed information. This research received grants from Medical Scientific Research Foundation of Zhejiang Province, China (2023XY002).

## Author contributions

**Conceptualization:** Yunyan Lu, Tian Lan.

**Formal analysis:** Yunyan Lu, Jing Zhang, Tian Lan, Jiawei He.

**Supervision:** Tian Lan, Jiawei He.

**Visualization:** Yunyan Lu, Tian Lan.

**Writing – original draft:** Yunyan Lu, Tian Lan.

**Writing – review & editing:** Yunyan Lu, Jing Zhang, Tian Lan, Jiawei He.

## Supplementary Material





## References

[1] Ruopp NF Cockrill BA. Diagnosis and treatment of pulmonary arterial hypertension: a review. JAMA. 2022;327:1379–91. 35412560 10.1001/jama.2022.4402

[2] Hassoun PM. Pulmonary arterial hypertension. N Engl J Med. 2021;385:2361–76. 34910865 10.1056/NEJMra2000348

[3] Pitre T Su J Cui S. Medications for the treatment of pulmonary arterial hypertension: a systematic review and network meta-analysis. Eur Respir Rev. 2022;31:220036. 35948391 10.1183/16000617.0036-2022 PMC9724821

[4] Escribano-Subias P Blanco I Lopez-Meseguer M.; REHAP investigators. Survival in pulmonary hypertension in Spain: insights from the Spanish registry. Eur Respir J. 2012;40:596–603. 22362843 10.1183/09031936.00101211

[5] Badesch DB Raskob GE Elliott CG. Pulmonary arterial hypertension: baseline characteristics from the REVEAL Registry. Chest. 2010;137:376–87. 19837821 10.1378/chest.09-1140

[6] Humbert M Sitbon O Chaouat A. Pulmonary arterial hypertension in France: results from a national registry. Am J Respir Crit Care Med. 2006;173:1023–30. 16456139 10.1164/rccm.200510-1668OC

[7] Jiang X Jing ZC. Epidemiology of pulmonary arterial hypertension. Curr Hypertens Rep. 2013;15:638–49. 24114080 10.1007/s11906-013-0397-5

[8] Rich S Haworth SG Hassoun PM Yacoub MH. Pulmonary hypertension: the unaddressed global health burden. Lancet Respir Med. 2018;6:577–9. 30072105 10.1016/S2213-2600(18)30268-6

[9] GBD 2021 Causes of Death Collaborators. Global burden of 288 causes of death and life expectancy decomposition in 204 countries and territories and 811 subnational locations, 1990-2021: a systematic analysis for the Global Burden of Disease Study 2021. Lancet. 2024;403:2100–32. 38582094 10.1016/S0140-6736(24)00367-2 PMC11126520

[10] Taichman DB Mandel J. Epidemiology of pulmonary arterial hypertension. Clin Chest Med. 2013;34:619–37. 24267294 10.1016/j.ccm.2013.08.010

[11] McGoon MD Benza RL Escribano-Subias P. Pulmonary arterial hypertension: epidemiology and registries. J Am Coll Cardiol. 2013;62(25 Suppl):D51–59. 24355642 10.1016/j.jacc.2013.10.023

[12] Rosenzweig EB Abman SH Adatia I. Paediatric pulmonary arterial hypertension: updates on definition, classification, diagnostics and management. Eur Respir J. 2019;53:1801916. 30545978 10.1183/13993003.01916-2018 PMC6351335

[13] Copher R Cerulli A Watkins A Laura Monsalvo M. Treatment patterns and healthcare system burden of managed care patients with suspected pulmonary arterial hypertension in the United States. J Med Econ. 2012;15:947–55. 22554140 10.3111/13696998.2012.690801

[14] Kirson NY Birnbaum HG Ivanova JI Waldman T Joish V Williamson T. Excess costs associated with patients with pulmonary arterial hypertension in a US privately insured population. Appl Health Econ Health Policy. 2011;9:293–303. 21875160 10.2165/11592430-000000000-00000

[15] Hoeper MM Humbert M Souza R. A global view of pulmonary hypertension. Lancet Respir Med. 2016;4:306–22. 26975810 10.1016/S2213-2600(15)00543-3

[16] Sikirica M Iorga SR Bancroft T Potash J. The economic burden of pulmonary arterial hypertension (PAH) in the US on payers and patients. BMC Health Serv Res. 2014;14:676. 25539602 10.1186/s12913-014-0676-0 PMC4301626

[17] Simonneau G Montani D Celermajer DS. Haemodynamic definitions and updated clinical classification of pulmonary hypertension. Eur Respir J. 2019;53:1801913. 30545968 10.1183/13993003.01913-2018 PMC6351336

[18] Vazquez ZGS Klinger JR. Guidelines for the treatment of pulmonary arterial hypertension. Lung. 2020;198:581–96. 32671468 10.1007/s00408-020-00375-w

[19] Okano JT Sharp K Valdano E Palk L Blower S. HIV transmission and source-sink dynamics in sub-Saharan Africa. Lancet HIV. 2020;7:e209–14. 32066532 10.1016/S2352-3018(19)30407-2 PMC7259817

[20] McManus DP Dunne DW Sacko M Utzinger J Vennervald BJ Zhou XN. Schistosomiasis. Nat Rev Dis Primers. 2018;4:13. 30093684 10.1038/s41572-018-0013-8

[21] Benza RL Farber HW Frost A. REVEAL risk scores applied to riociguat-treated patients in PATENT-2: impact of changes in risk score on survival. J Heart Lung Transplant. 2018;37:513–9. 29223470 10.1016/j.healun.2017.11.006

[22] Galie N Humbert M Vachiery JL. 2015 ESC/ERS Guidelines for the diagnosis and treatment of pulmonary hypertension: the joint task force for the diagnosis and treatment of pulmonary hypertension of the European Society of Cardiology (ESC) and the European Respiratory Society (ERS): endorsed by: Association for European Paediatric and Congenital Cardiology (AEPC), International Society for Heart and Lung Transplantation (ISHLT). Eur Respir J. 2015;46:903–75. 26318161 10.1183/13993003.01032-2015

[23] Budhiraja R Tuder RM Hassoun PM. Endothelial dysfunction in pulmonary hypertension. Circulation. 2004;109:159–65. 14734504 10.1161/01.CIR.0000102381.57477.50

[24] Hasan B Hansmann G Budts W.; European Pediatric Pulmonary Vascular Disease Network (EPPVDN), endorsed by AEPC, CSC, PASCAR, PCS, and PCSI. Challenges and special aspects of pulmonary hypertension in middle- to low-income regions: JACC state-of-the-art review. J Am Coll Cardiol. 2020;75:2463–77. 32408981 10.1016/j.jacc.2020.03.047

[25] Boucly A Gerges C Savale L. Pulmonary arterial hypertension. Presse Med. 2023;52:104168. 37516248 10.1016/j.lpm.2023.104168

[26] GBD 2021 Diseases and Injuries Collaborators. Global incidence, prevalence, years lived with disability (YLDs), disability-adjusted life-years (DALYs), and healthy life expectancy (HALE) for 371 diseases and injuries in 204 countries and territories and 811 subnational locations, 1990-2021: a systematic analysis for the Global Burden of Disease Study 2021. Lancet. 2024;403:2133–61. 38642570 10.1016/S0140-6736(24)00757-8 PMC11122111

