# Supplementary material for: Global, regional, and national burden of pulmonary arterial hypertension from 1990 to 2021
Source: Medicine (Baltimore). 2025 Oct 10;104(41):e44933. doi: 10.1097/MD.0000000000044933 (PMC12517792; doi:10.1097/MD.0000000000044933)
Supplement: Supplementary file 2 [file medi-104-e44933-s002.pdf]

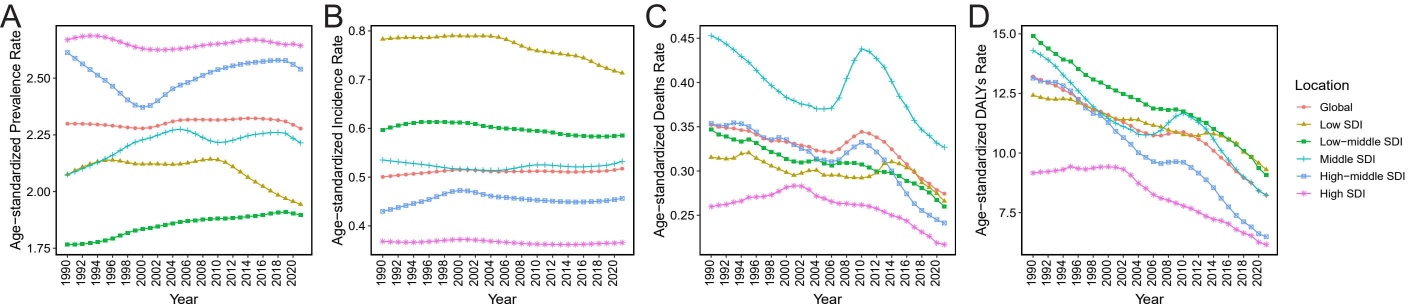

A
B
C
D
Age-standardized Prevalence Rate
Age-standardized Incidence Rate
Age-standardized Deaths Rate
Age-standardized DALYs Rate
Year
Location
Global
Low SDI
Low-middle SDI
Middle SDI
High-middle SDI
High SDI

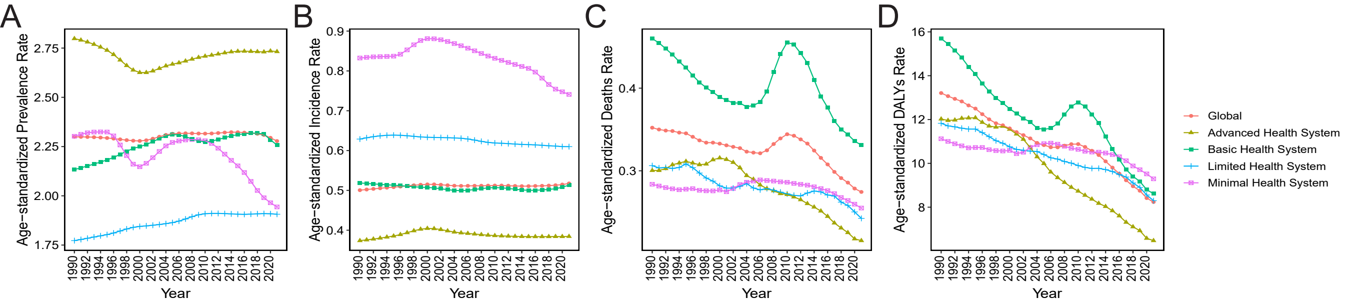

A
B
C
D
Age-standardized Prevalence Rate
Age-standardized Incidence Rate
Age-standardized Deaths Rate
Age-standardized DALYs Rate
Year
Global
Advanced Health System
Basic Health System
Limited Health System
Minimal Health System

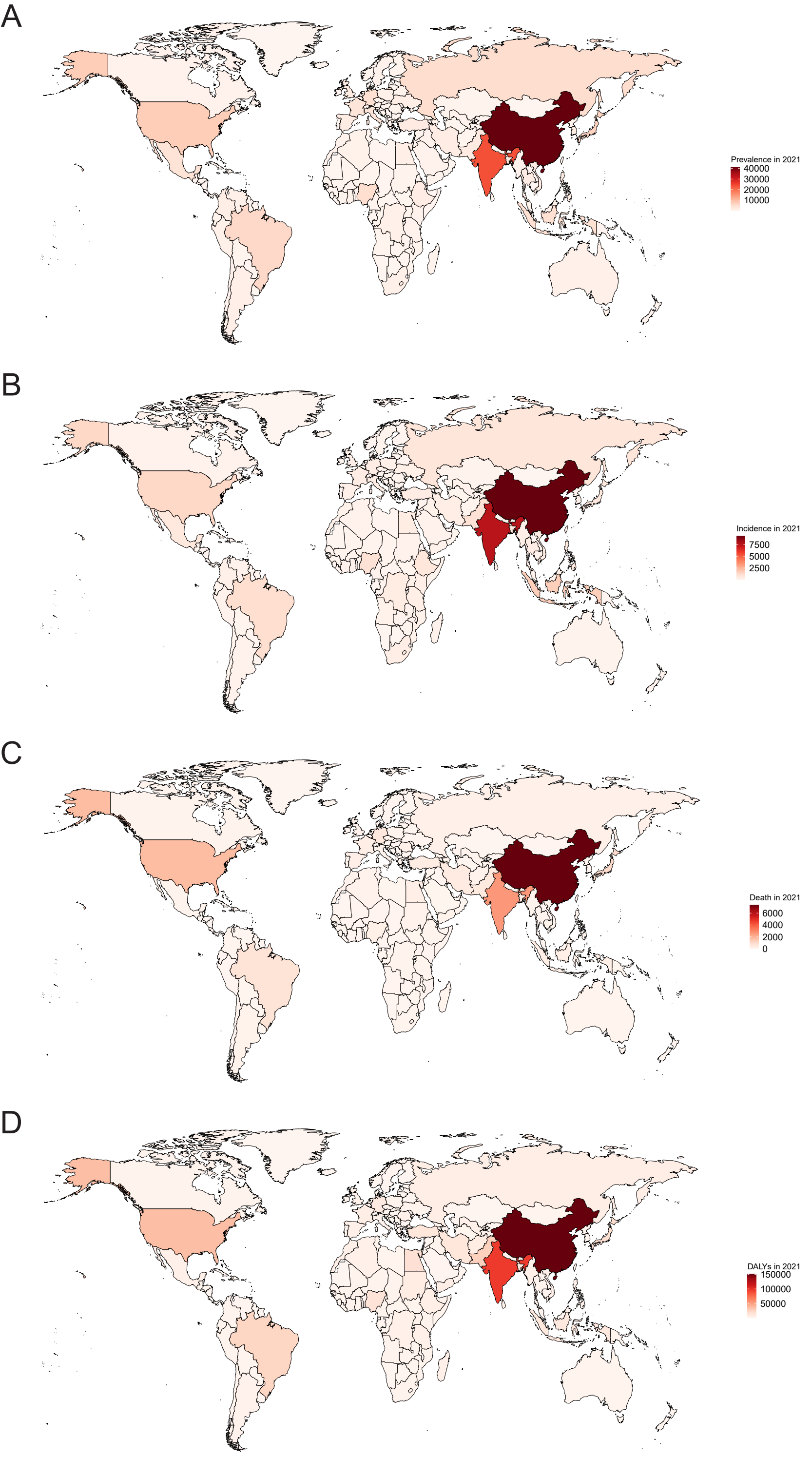
A
Prevalence in 2021
40000
30000
20000
10000
B
Incidence in 2021
7500
5000
2500
C
Death in 2021
6000
4000
2000
0
D
DALYs in 2021
150000
100000
50000

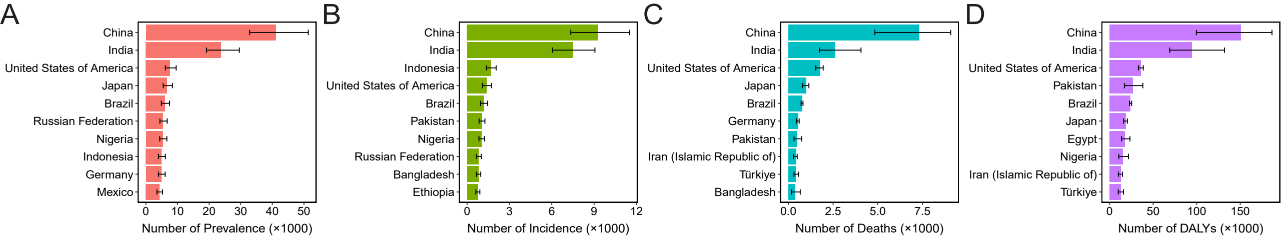

A
China
India
United States of America
Japan
Brazil
Russian Federation
Nigeria
Indonesia
Germany
Mexico
0
10
20
30
40
50
Number of Prevalence (×1000)
B
China
India
Indonesia
United States of America
Brazil
Pakistan
Nigeria
Russian Federation
Bangladesh
Ethiopia
0
3
6
9
12
Number of Incidence (×1000)
C
China
India
United States of America
Japan
Brazil
Germany
Pakistan
Iran (Islamic Republic of)
Türkiye
Bangladesh
0.0
2.5
5.0
7.5
Number of Deaths (×1000)
D
China
India
United States of America
Pakistan
Brazil
Japan
Egypt
Nigeria
Iran (Islamic Republic of)
Türkiye
0
50
100
150
Number of DALYs (×1000)

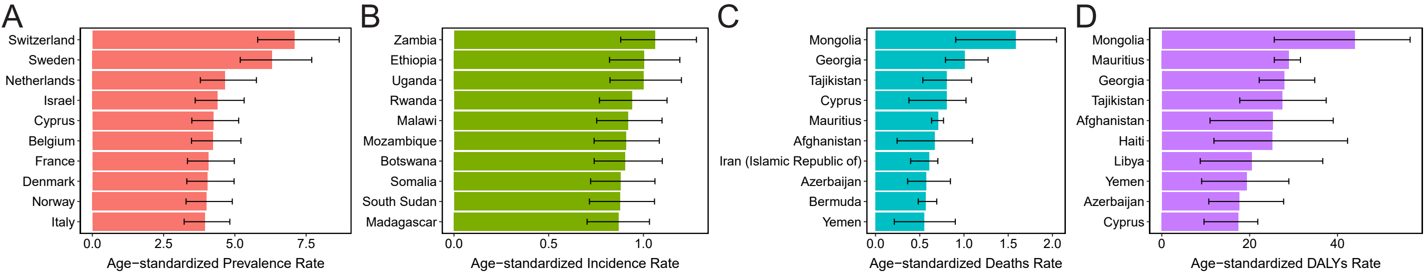

A
Switzerland
Sweden
Netherlands
Israel
Cyprus
Belgium
France
Denmark
Norway
Italy
0.0
2.5
5.0
7.5
Age−standardized Prevalence Rate
B
Zambia
Ethiopia
Uganda
Rwanda
Malawi
Mozambique
Botswana
Somalia
South Sudan
Madagascar
0.0
0.5
1.0
Age−standardized Incidence Rate
C
Mongolia
Georgia
Tajikistan
Cyprus
Mauritius
Afghanistan
Iran (Islamic Republic of)
Azerbaijan
Bermuda
Yemen
0.0
0.5
1.0
1.5
2.0
Age−standardized Deaths Rate
D
Mongolia
Mauritius
Georgia
Tajikistan
Afghanistan
Haiti
Libya
Yemen
Azerbaijan
Cyprus
0
20
40
Age−standardized DALYs Rate

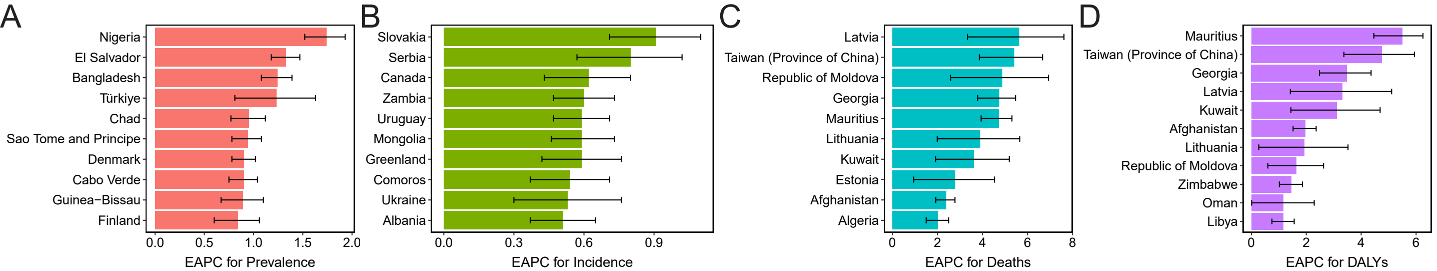
A
Nigeria
El Salvador
Bangladesh
Türkiye
Chad
Sao Tome and Principe
Denmark
Cabo Verde
Guinea−Bissau
Finland
0.0 0.5 1.0 1.5 2.0
EAPC for Prevalence
B
Slovakia
Serbia
Canada
Zambia
Uruguay
Mongolia
Greenland
Comoros
Ukraine
Albania
0.0 0.3 0.6 0.9
EAPC for Incidence
C
Latvia
Taiwan (Province of China)
Republic of Moldova
Georgia
Mauritius
Lithuania
Kuwait
Estonia
Afghanistan
Algeria
0 2 4 6 8
EAPC for Deaths
D
Mauritius
Taiwan (Province of China)
Georgia
Latvia
Kuwait
Afghanistan
Lithuania
Republic of Moldova
Zimbabwe
Oman
Libya
0 2 4 6
EAPC for DALYs

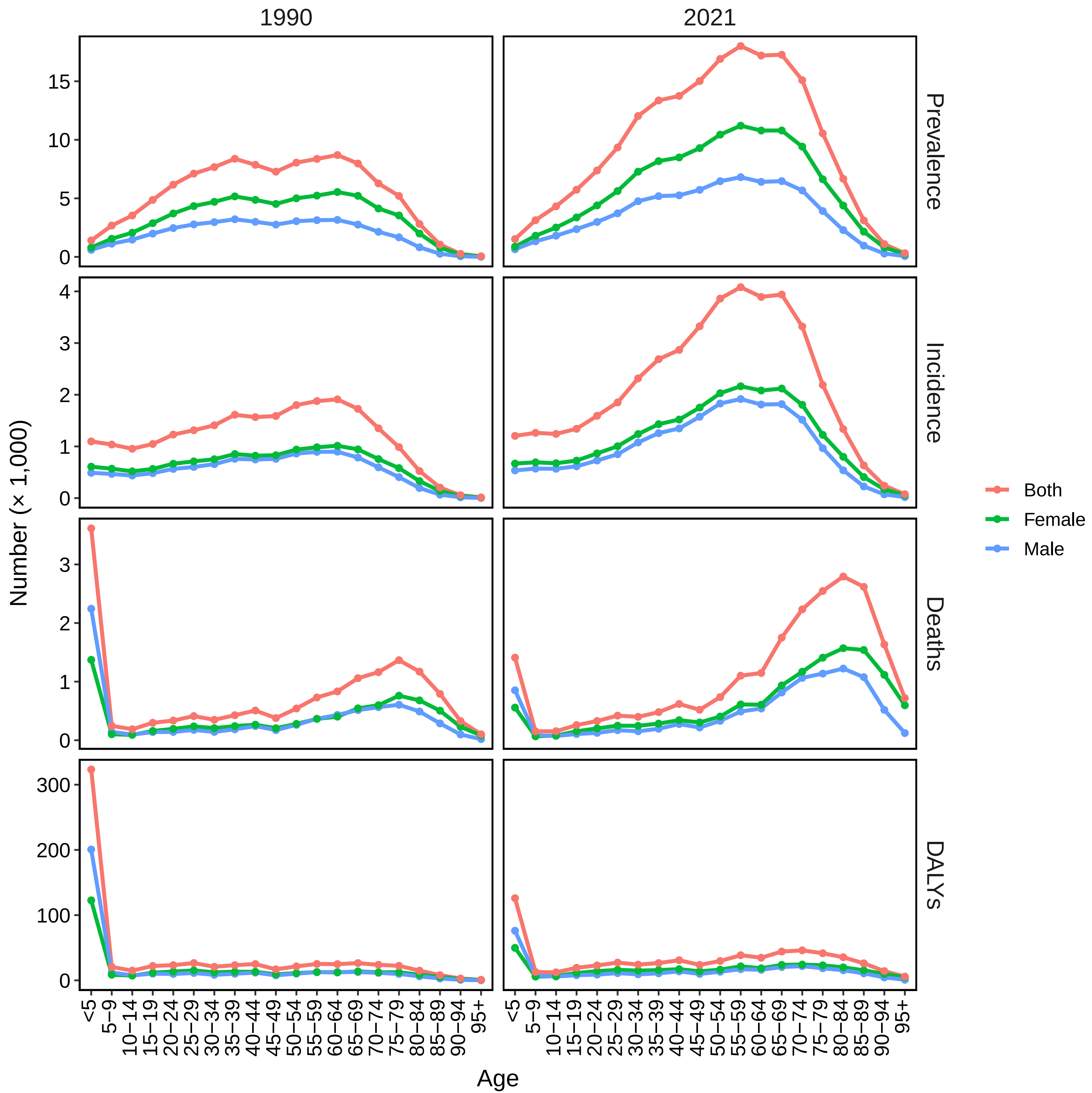

1990
2021
Prevalence
Incidence
Deaths
DALYs
Number (× 1,000)
Age
<5
5–9
10–14
15–19
20–24
25–29
30–34
35–39
40–44
45–49
50–54
55–59
60–64
65–69
70–74
75–79
80–84
85–89
90–94
95+
Both
Female
Male

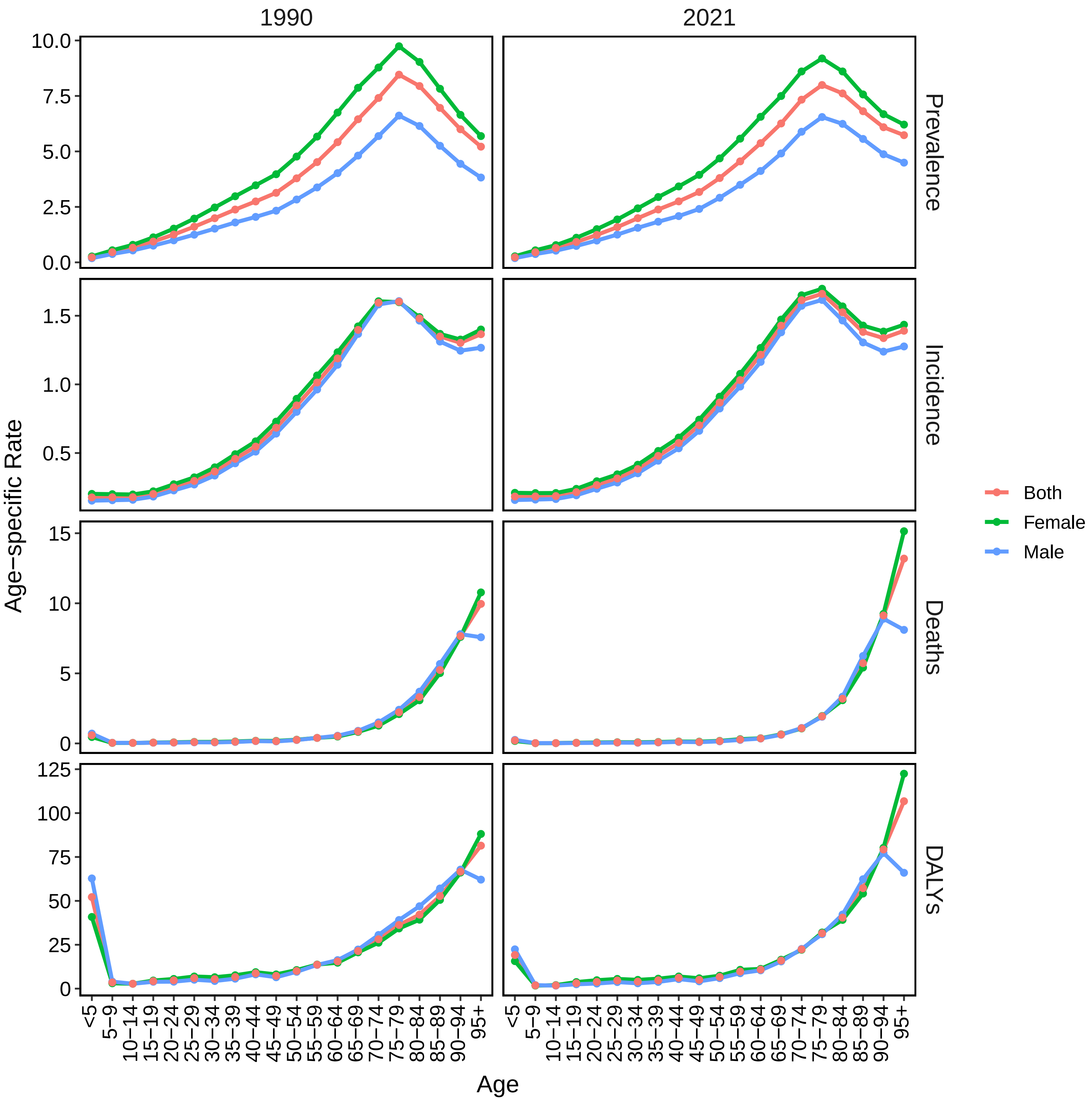

1990
2021
Prevalence
Incidence
Deaths
DALYs
Age−specific Rate
Age
Both
Female
Male
<5
5−9
10−14
15−19
20−24
25−29
30−34
35−39
40−44
45−49
50−54
55−59
60−64
65−69
70−74
75−79
80−84
85−89
90−94
95+

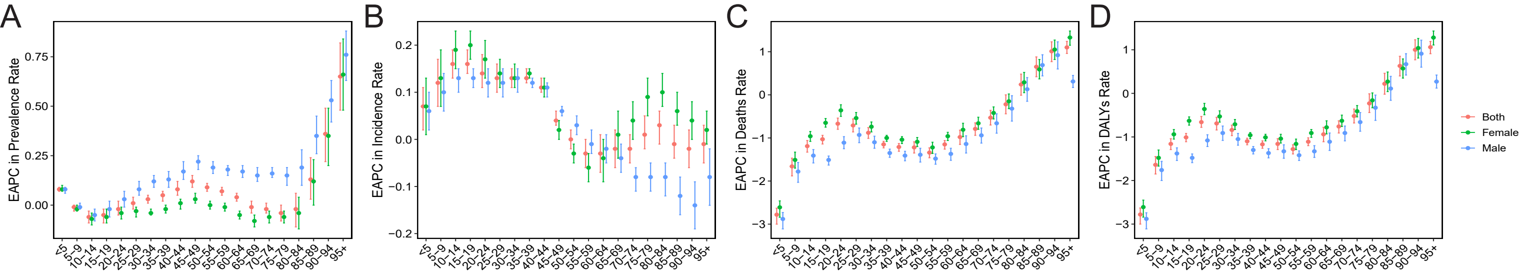

A
EAPC in Prevalence Rate
0.75
0.50
0.25
0.00
B
EAPC in Incidence Rate
0.2
0.1
0.0
−0.1
−0.2
C
EAPC in Deaths Rate
1
0
−1
−2
−3
D
EAPC in DALYs Rate
1
0
−1
−2
−3
<5
5–9
10–14
15–19
20–24
25–29
30–34
35–39
40–44
45–49
50–54
55–59
60–64
65–69
70–74
75–79
80–84
85–89
90–94
95+
Both
Female
Male

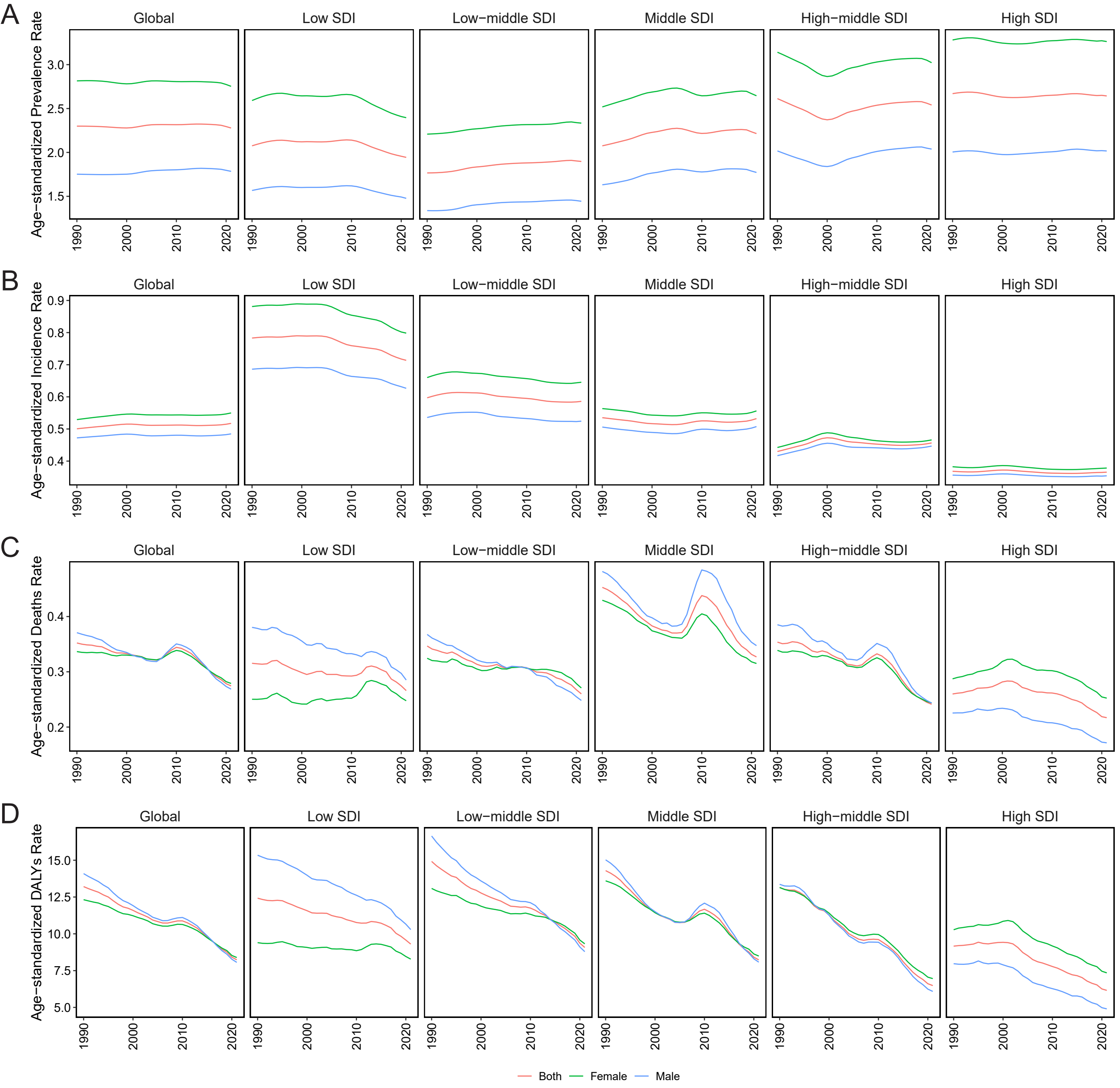

A
B
C
D
Global
Low SDI
Low−middle SDI
Middle SDI
High−middle SDI
High SDI
Age−standardized Prevalence Rate
Age−standardized Incidence Rate
Age−standardized Deaths Rate
Age−standardized DALYs Rate
1990
2000
2010
2020
Both
Female
Male

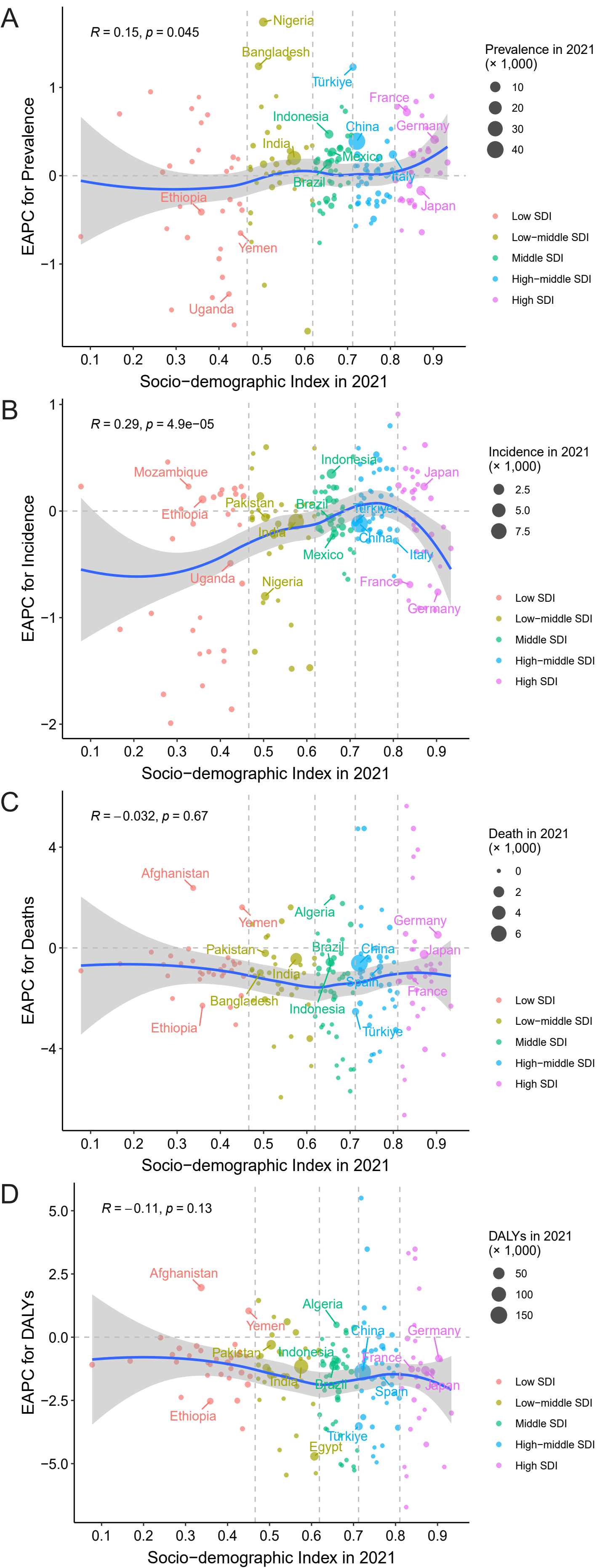
A
R = 0.15, p = 0.045
EAPC for Prevalence
Socio−demographic Index in 2021
Nigeria
Bangladesh
Türkiye
France
Germany
Indonesia
China
India
Mexico
Italy
Brazil
Ethiopia
Japan
Yemen
Uganda
Prevalence in 2021
(× 1,000)
10
20
30
40
Low SDI
Low−middle SDI
Middle SDI
High−middle SDI
High SDI
B
R = 0.29, p = 4.9e−05
EAPC for Incidence
Socio−demographic Index in 2021
Indonesia
Mozambique
Japan
Pakistan
Brazil
Türkiye
Ethiopia
India
China
Mexico
Italy
Uganda
Nigeria
France
Germany
Incidence in 2021
(× 1,000)
2.5
5.0
7.5
Low SDI
Low−middle SDI
Middle SDI
High−middle SDI
High SDI
C
R = −0.032, p = 0.67
EAPC for Deaths
Socio−demographic Index in 2021
Afghanistan
Yemen
Algeria
Germany
Pakistan
Brazil
China
Japan
India
Spain
France
Bangladesh
Indonesia
Ethiopia
Türkiye
Death in 2021
(× 1,000)
0
2
4
6
Low SDI
Low−middle SDI
Middle SDI
High−middle SDI
High SDI
D
R = −0.11, p = 0.13
EAPC for DALYs
Socio−demographic Index in 2021
Afghanistan
Algeria
Yemen
China
Germany
Pakistan
Indonesia
France
India
Brazil
Japan
Spain
Ethiopia
Türkiye
Egypt
DALYs in 2021
(× 1,000)
50
100
150
Low SDI
Low−middle SDI
Middle SDI
High−middle SDI
High SDI
